# HSP90 Interacts with the Fibronectin N-terminal Domains and Increases Matrix Formation

**DOI:** 10.3390/cells9020272

**Published:** 2020-01-22

**Authors:** Abir Chakraborty, Natasha Marie-Eraine Boel, Adrienne Lesley Edkins

**Affiliations:** 1 Biomedical Biotechnology Research Unit, Department of Biochemistry and Microbiology, Rhodes University, Grahamstown 6140, South Africa; abir216gogol@gmail.com (A.C.); natashaboel@gmail.com (N.M.-E.B.); 2 Centre for Chemico- and Biomedicinal Research, Rhodes University, Grahamstown 6140, South Africa

**Keywords:** HSP90, fibronectin, extracellular matrix, client protein, fibrillogenesis

## Abstract

Heat shock protein 90 (HSP90) is an evolutionarily conserved chaperone protein that controls the function and stability of a wide range of cellular client proteins. Fibronectin (FN) is an extracellular client protein of HSP90, and exogenous HSP90 or inhibitors of HSP90 alter the morphology of the extracellular matrix. Here, we further characterized the HSP90 and FN interaction. FN bound to the M domain of HSP90 and interacted with both the open and closed HSP90 conformations; and the interaction was reduced in the presence of sodium molybdate. HSP90 interacted with the N-terminal regions of FN, which are known to be important for matrix assembly. The highest affinity interaction was with the 30-kDa (heparin-binding) FN fragment, which also showed the greatest colocalization in cells and accommodated both HSP90 and heparin in the complex. The strength of interaction with HSP90 was influenced by the inherent stability of the FN fragments, together with the type of motif, where HSP90 preferentially bound the type-I FN repeat over the type-II repeat. Exogenous extracellular HSP90 led to increased incorporation of both full-length and 70-kDa fragments of FN into fibrils. Together, our data suggested that HSP90 may regulate FN matrix assembly through its interaction with N-terminal FN fragments.

## 1. Introduction

The 90-kDa ATP-driven chaperone family protein, heat shock protein 90 (HSP90), is widely distributed in eukaryotes, and creates a proteostasis hub inside the cell [1,2,3,4]. HSP90 is mostly found in the cytoplasm and constitutes almost 2% of the cellular protein content [5,6,7]. Each monomer of the HSP90 dimer has three domains, namely an N-terminal domain (NTD) that binds and hydrolyses ATP, a middle domain (MD/M domain) connected to the NTD via a charged linker, and a carboxy (C)-terminal domain that is vital for dimerization and possesses the tetratricopeptide repeat (TPR)-binding motif, EEVD [4,8,9,10,11,12]. The M domain is believed to be the major client-binding site and discriminates between different types of client proteins [13,14,15]. The charged linker which connects the NTD to the MD operates in client activation and co-chaperone binding [6,15,16,17]. HSP90 regulates the stability of a substantial proportion of the cellular proteome, including numerous signaling intermediates, kinases, and transcription factors [2,7,18,19]. HSP90 function is best characterized in the cytoplasm, although it is now clear that HSP90 is also exported to the extracellular space where it functions in the regulation of the immune response and cellular processes like migration and invasion [3,20,21,22,23,24]. In this way, extracellular HSP90 (exHSP90) potentiates the aggressiveness of cancer by promoting cancer progression and metastasis [20]. Compared to the wide range of intracellular clients, fewer exHSP90 clients have been identified, but those that have been identified include a number of extracellular matrix-associated proteins like matrix metalloproteases (MMP2 and MMP9), tissue plasminogen activator (TPA), and fibronectin, which provide a mechanistic link for the role of exHSP90 in migration and invasion [22,25,26,27]. Current dogma suggests that HSP90 identifies client proteins through recognition of unstable conformations, rather than sequence-based motifs [28,29]. Compared to intracellular client proteins, a relatively few studies have studied the interaction of HSP90 with extracellular client proteins in any depth.

Our previous work identified the extracellular matrix protein fibronectin (FN) as a client protein of HSP90, and demonstrated a role for extracellular HSP90 in regulating matrix assembly and turnover via the extracellular receptor low density lipoprotein receptor-related protein-1 (LRP1/CD91) [30,31]. Tumors with a high level of FN expression are associated with a higher chance of metastasis, and elevated HSP90 and FN are correlated with decreased survival in breast cancer patients [32,33,34,35,36]. N-terminal inhibition of HSP90 with either geldanamycin or AUY922 increased FN expression, while AUY922 reduced FN secretion in prostate cancer which was linked to a reduction in invasion and migration [37]. Beyond cancer, increased expression of FN and extracellular matrix (ECM) are associated with idiopathic pulmonary fibrosis (IPF) [38]. Elevated extracellular HSP90α levels were associated with increased severity of IPF, where HSP90α secretion increased with increased matrix stiffness [38]. Therefore, HSP90 modulation may provide a mechanism by which to treat disorders associated with dysregulated FN and ECM. In addition to therapeutic potential, the regulation of FN ECM is also interesting from a protein folding perspective, since FN is synthesized and exported in a soluble form, which subsequently undergoes cell-mediated unfolding and self-association (in addition to numerous other interactions) to form an insoluble extracellular matrix [39,40,41,42,43]. This process involves substantial protein conformational regulation [41,44,45], and hence it is not surprising that HSP90 may play a role. The mechanism by which the HSP90–FN interaction regulates FN assembly or turnover remains undefined. Part of defining that mechanism is our current study aimed at understanding the molecular basis for the interaction between HSP90 and FN in more detail.

## 2. Materials and Methods

### 2.1. Proteins, Antibodies, and Plasmids

**Figure 1:**
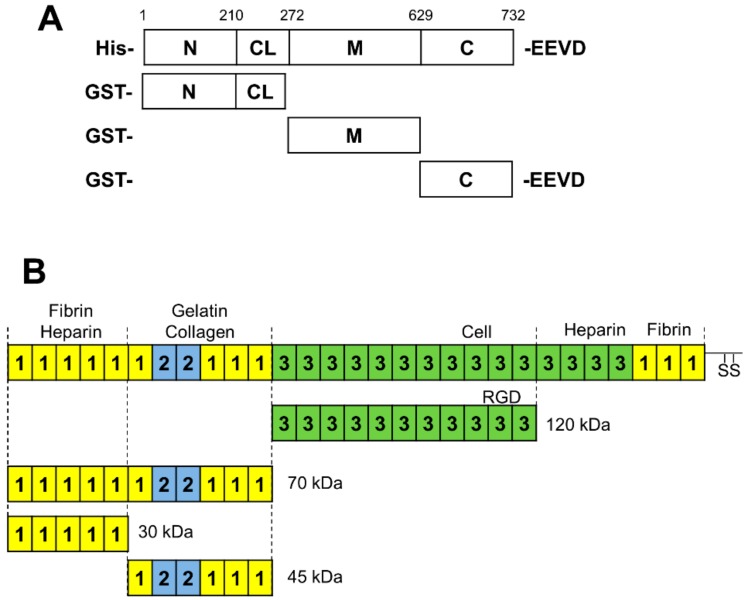
Schematic diagram of HSP90 and fibronectin (FN) domains. (**A**) HSP90 domain boundaries indicated by numbering and recombinant fragments used in this study. (**B**) Domain structure of full-length fibronectin and proteolytic fragments thereof. The squares labeled 1, 2, and 3 refer to the type-I, type-II, and type-III FN domains, respectively. The binding sites of FN interactors are labeled above, while the sites of proteolytic cleavage of full-length FN are indicated by dotted lines and they give rise to the smaller 120, 70, 45, and 30 kDa fragments used in this study.

The 120-kDa cell-binding FN fragment (henceforth referred to as FN120) was from Merck Millipore (F1904, Burlington, MA, United States) and full-length FN (FL-FN) was from Santa Cruz Biotechnology (SC29011, Dallas, TX, United States). The 70-kDa N-terminal FN fragment produced by cathepsin D treatment of full-length FN (^1–6^FNI^1–2^FNII^7–9^FNI; henceforth referred to as FN70) (F0287); and the N-terminal FN 30-kDa heparin-binding fragment (^1–5^FN1, henceforth referred to as FN30) (F9911) and the 45-kDa gelatin-binding FN fragment (^6^FNI^1–2^FNII^7–9^FNI, henceforth referred to as FN45) (F0162) produced by tryptic cleavage of the 70-kDa fragment were from Sigma-Aldrich (St. Louis, MO, United States) (Figure 1). The details of all antibodies, together with conditions of usage in the study are provided in the Supplementary Files. Recombinant full-length His-HSP90α and the glutathione S-transferase (GST)-tagged HSP90α domains (Figure 1) were purified from *Escherichia coli* using established protocols, the detail of which can be found in the Supplementary Files.

### 2.2. Plasmids

pGEX-4T-1-GST-HSP90M (Addgene plasmid #22482; http://n2t.net/addgene:22482; RRID: Addgene_22482), pGEX-4T-1-GST-HSP90C (Addgene plasmid #22483; http://n2t.net/addgene:22483; RRID: Addgene_22483), and pGEX-4T-1-GST-HSP90N (Addgene plasmid #22481; http://n2t.net/addgene:22481; RRID: Addgene_22481) were a gift from William Sessa [46]. pHLSec2-FN-YPet (Addgene plasmid #65421; http://n2t.net/addgene:65421; RRID: Addgene_65421) was a gift from Harold Erickson [47]. pBiFC-VC155 (Addgene plasmid #22011; http://n2t.net/addgene:22011; RRID: Addgene_22011), pBiFC-VN173 (Addgene plasmid #22010; http://n2t.net/addgene:22010; RRID: Addgene_22010), pBiFC-bfosVC155 (Addgene plasmid #22013; http://n2t.net/addgene:22013; RRID: Addgene_22013), and pBiFC-bJunVN173 (Addgene plasmid #22012; http://n2t.net/addgene:22012; RRID: Addgene_22012) were a gift from Chang-Deng Hu [48]. pCherry.90beta (Addgene plasmid #108223; http://n2t.net/addgene:108223; RRID: Addgene_108223) was a gift from Didier Picard [49]. pcDNA-Flag-HSP90α-WT, pcDNA-Flag-HSP90α-Y313E/F, pcDNA-HA-HSP90α-WT, and pcDNA-HA-HSP90α-E47A were a gift from Len Neckers [50,51]. pcDNA-Flag-HSP90α-D93A was a gift from Mehdi Mollapour [52].

The coding sequences of FN30 and FN70 including the signal sequence were cloned into pBiFC-VC155 in-frame with a haemagglutinin (HA) tag via the *Apa*I site, whereas HSP90α and HSP90αM were cloned into the pBiFC-VN173 plasmid in-frame with a Flag tag via the *Bgl*II/*Sal*I and *Sal*I site, respectively. For enhanced green fluorescence protein (EGFP)-fusion FN fragment-expressing plasmids, the coding sequences of FN70 and FN30 were cloned into pcDNA3-EGFP via the *Bam*HI/*Eco*RI sites in-frame with C-terminal EGFP. The FN short repeats encoding either 3 type-I repeats (^1–3^FNI) or 2 type-II repeats (^1–2^FNII) were synthesized by GenScript (Hong Kong) with C-terminal HA and hexahistidine (His) tags, and were subcloned into the pcDNA3 plasmid via the *Bam*HI/*Eco*RI sites. The His-HSP90α-M domain was constructed by subcloning from pGEX-4T-1-GST-HSP90M into pQE80L in-frame with an N-terminal His tag at *Bam*HI/*Sal*I site. The codon-optimized HSP90α-coding region was synthesized and inserted into the pET16b expression plasmid in-frame with an N-terminal His tag by GenScript (Hong Kong). Further details on PCR parameters, primers, recombinant protein expression, and purification protocols can be found in the Supplementary Files.

### 2.3. Cell Line Maintenance and Transfection

The HEK293T cell line was maintained in Dulbecco’s modified Eagle’s medium (DMEM) with 10% (*v/v*) fetal bovine serum (FBS), 1% (*v/v*) L-glutamine, 0.1 mM non-essential amino acids (NEAA), 1 mM sodium pyruvate, 100 U/mL penicillin, streptomycin and amphotericin (PSA), and 500 µg/mL G418. Hs578T breast cancer cells were maintained in DMEM supplemented with 10% FBS, 2 mM GlutaMAX™, 100 U/mL PSA, and 2 mM insulin (Novo Nordisk A/S, Bagsværd, Denmark). Mouse embryonic fibroblasts (MEFs) were maintained in DMEM supplemented with 10% (*v/v*) FBS, 2 mM GlutaMAX™, and 100 U/mL PSA. All cell lines were maintained at 37 °C in a humidified chamber with 9% CO_2_. Cells were transfected at 50% confluency with X-tremeGENE HP DNA transfection reagent (Roche, Manheim, Germany; 6366244001) according to the protocol provided.

### 2.4. Sodium Dodecyl Sulphate–Polyacrylamide Gel Electrophoresis (SDS-PAGE) and Western Blot Analysis

Cells were lysed in CelLytic^TM^ M (Sigma-Aldrich, St Louis, MO, USA) and total protein quantified was using absorbance at 280 nm on a Nanodrop 2000 spectrophotometer. Equal amounts of protein were loaded into each well and separated in discontinuous SDS-PAGE according to the accepted modified protocol of Laemmli (1970) [53]. Resolved proteins were transferred onto a nitrocellulose membrane for western blot analysis using the method of Towbin and Gordon (1979) [54]. For the list of antibodies and dilutions used in this study, please see the Supplementary Materials.

### 2.5. Solid Phase Protein–Protein Interaction Assay

Solid phase protein–protein interaction assays were performed according to the published protocol [30]. Briefly, high binding plates were coated with 100 µg/mL of either full-length FN or FN30, FN45, FN70, or FN120 fragments, and incubated for 6 h at 22 °C before blocking with 3% (*w/v*) bovine serum albumin (BSA) in Buffer A [30]. The interacting protein (HSP90α or HSP90α N, M or C domain) was added and incubated overnight at 4 °C. Plates were washed three times with 1% (*w/v*) BSA in Buffer A and treated with primary antibody in Buffer A. Plates were washed with 1% (*w/v*) BSA and incubated with the appropriate horseradish peroxidase (HRP)-conjugated secondary antibody solution. A 3,3′,5,5′, tetramethylbenzidine TMB substrate solution (0.1 mg/mL TMB in 25.7 mM citric acid, 48.6 mM disodium phosphate, and 0.01% (*v/v*) hydrogen peroxide) was added to each well and the reaction was stopped by adding 2 M sulfuric acid. Absorbance values were recorded at 450 nm and data were processed using GraphPad Prism software version 4.0.

### 2.6. Gelatin and Heparin Agarose Pull-Down Assay

A 20 µL aliquot of either gelatin agarose or heparin agarose beads was incubated with a dose-dependent amount (0, 1, 5, and 50 µg) of purified His-HSP90α protein. Each bead–protein mixture was mixed with 200 µg VC-FN70-transfected cell lysates in a 600 µL total reaction volume in phosphate buffered saline (PBS), and incubated overnight at 4 °C with rotation. PBS-T (PBS containing 0.05% (*v/v*) Tween 20) with 50 mM phenylmethanesulfonyl fluoride (PMSF) was used to wash the beads, before eluting the complex by boiling in SDS loading buffer with 5% (*v/v*) β-mercaptoethanol and analyzing the complex by SDS-PAGE and western blot.

### 2.7. Immunoprecipitation and Nickel Affinity Pull-Down Assay

Transfected mammalian cell lysates were incubated with anti-EGFP or anti-HA conjugated beads overnight at 4 °C with shaking. Beads were washed with PBS-T (0.05% (*v/v*) Tween 20 with 50 mM PMSF) for three times. Subsequently, protein complexes were eluted by boiling in SDS loading buffer for 5 min. For isolation of His-tagged proteins from transfected cells, cell lysates were incubated with nickel conjugated nitrilotriacetic acid (Ni-NTA) beads overnight at 4 °C. Ni-NTA agarose beads were washed with a His wash buffer (20 mM Tris-HCl (pH 7.5), 100 mM NaCl, 20 mM imidazole, and 50 mM PMSF). Proteins were eluted by boiling in the SDS loading buffer with β-mercaptoethanol and analyzed by western blot.

### 2.8. Protein Thermal Shift Unfolding Assay

Thermal scanning of protein unfolding was adapted from published protocols using the CFX Connect thermal cycler system (BioRad, Hercules, CA, United States) [55,56]. For the unfolding assay, a 5x final concentration of SYPRO Orange dye was mixed with 2 µM protein in a 20 µL reaction volume in standard 25 mM sodium phosphate buffer. A stepwise temperature increment (0.7 °C) from 25 °C to 95 °C with 3 min hold times and an initial 2 min hold time was applied and fluorescence signal was detected using a VIC filter set. The melting temperature (T_m_) of unfolding was generated from raw fluorescence values using GraphPad Prism 4.

### 2.9. Confocal Microscopy for Colocalization and Biomolecular Fluorescence Complementation (BiFC) Assay

HEK293T cells were seeded onto poly-L-lysine-coated glass coverslips and allowed to adhere overnight. For either colocalization analysis [30] or BiFC assay [48], cells were transfected with appropriate plasmids at 60% confluency. Following 48 h transfection, cells were fixed with freshly prepared 3.7% (*w/v*) paraformaldehyde and stained with 1 µg/mL Hoechst 33342 before mounting with DAKO mounting medium and analysis by confocal microscopy (Carl Zeiss, Jena, Germany).

### 2.10. Confocal Microscopy Analysis of FN Matrix

Protocols for ECM production and harvest from Hs578T breast carcinoma cells were adapted from published protocols [57]. Ethanol-sterilized coverslips in a 6-well plate were incubated with 0.2% (*w/v*) sterile gelatin for 1 h at 37 °C. Gelatin was crosslinked with 1% (*v/v*) sterile glutaraldehyde in PBS for 30 min at room temperature. Wells were washed thrice with PBS, 5 min each time. Crosslinker was quenched with 1 M sterile ethanolamine for 30 min at room temperature. Wells were again washed thrice with PBS, 5 min each time. Cells were seeded onto the prepared 6-well plates (at a density of 6 × 10^5^ cells/well or such that the next day 100% confluency was observed). Upon confluency, the media was replaced with 50 μg/mL ascorbic acid-containing media and was changed every second day thereafter. After 6 days of culture, wells were treated for 24 h with BSA or HSP90β as described in figure legends. The following day, cells were washed with PBS and incubated with 50 mM EDTA for 10 min at 37 °C. Cells were washed twice with PBS and then incubated with an extraction buffer (20 mM NH_4_OH and 0.5% (*v/v*) Triton-X in PBS) preheated to 37 °C until complete cell lysis (as observed by assessing whether all cells had lifted using an inverted light microscope). Without removing the extraction buffer, PBS was added to each of the wells and placed at 4 °C overnight to improve the stability of the newly extracted matrices. To remove DNA, wells were incubated with 10 μg/mL of DNase I (Roche, Basel, Switzerland) for 30 min at 37 °C. Wells were washed three times with PBS. Cell-derived matrices were processed for immunostaining and confocal microscopy as described previously [31].

For the treatment of Hs578T cells with fluorescently labeled FN and FN70, commercial sources of FL-FN and FN70 resuspended in PBS were fluorescently conjugated to a DyLight dye by an N-hydroxysuccinimide (NHS) ester moiety. FL-FN and FN70 were conjugated to DyLight 488 (Catalog number: 53025) and DyLight 555 (Catalog number: 53044), respectively, according to the manufacturer’s instructions. Hs578T cells were seeded on glass coverslips and grown for 48 h. FL-FN-488 was added to cells for 24 h. Cells were then left untreated or treated with either HSP90β or BSA, together with the addition of FN70-555, to the existing medium containing FL-FN-488 for the time periods indicated in figure legends. Coverslips were harvested at the various time points and fixed with 4% (*w/v*) paraformaldehyde. Nuclei were stained with Hoechst-33342 dye. Images were captured using the Zeiss LSM780 Meta laser scanning confocal microscope (Zeiss, Munich, Germany) and analyzed using the Zen Blue Software (Zen2, blue edition).

### 2.11. Cell Migration Assays

For the scratch assays, MEF-1 and Hs578T cells were plated in 24-well culture plates to form a confluent cell monolayer. Using a sterile toothpick, a scratch was made in the monolayer to create wounds. The wells were washed once with medium to remove non-adherent cells and incubated in fresh medium containing various HSP90 inhibitors as indicated in figure legends. Images were taken with a Zeiss Primovert inverted light microscope at the time of initiation of the wound (*t* = 0 h) and again after 12 h migration (*t* = 12 h). Distances migrated were calculated by subtracting the wound width at *t* = 12 h from the wound width at *t* = 0 h. For migration assays from a plated monolayer, cells were plated into 4-well culture inserts (ibidi, Lochhamar, Schlag 11|82166 Grafelfing, Germany; Catalog number: 80469) to achieve confluency. Cells were left untreated or treated with the HSP90 inhibitor, novobiocin, for 16 h. Inserts were removed and the migration of cells outward from the monolayer edges was measured by capturing images at the start (*t* = 0 h) and end of the 12 h migration (*t* = 12 h) period. The distance migrated was calculated by measuring the distance of migrating cell border from the original cell border.

### 2.12. Statistical Analysis and Reproducibility

All data represent a minimum of three independent experiments, unless otherwise stated. Statistical analysis using unpaired t-tests, one-way ANOVA, and two-way ANOVA with Bonferroni post-test were performed in GraphPad Prism 4 and a *p*-value below 0.05 was considered to be statistically significant.

## 3. Results

### 3.1. Identification of the Interaction Domains in HSP90 and FN

Structurally, HSP90 is composed of three domains which regulate its function. The N-terminal domain is required for ATP binding and hydrolysis, while the M domain is the primary binding site of client proteins and the C-terminal domain mediates dimerization and co-chaperone interactions (Figure 1).

**Figure 2:**
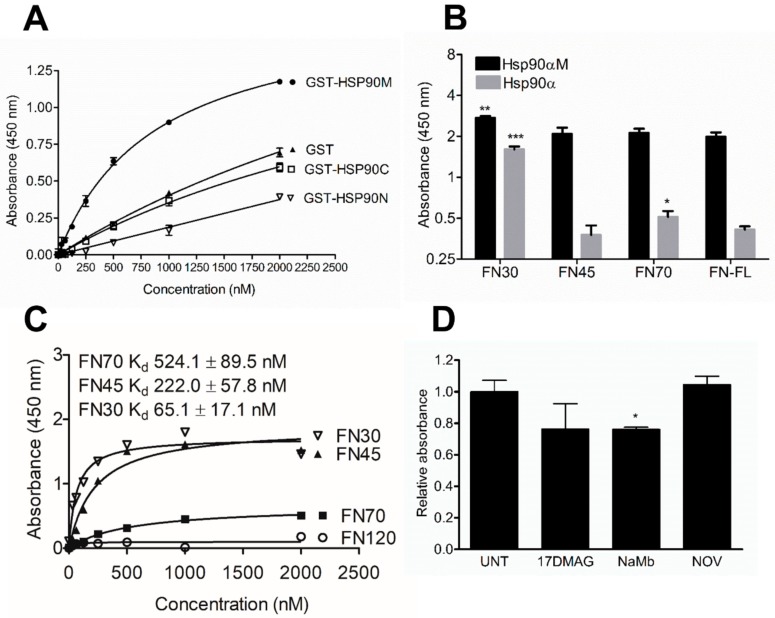
HSP90M and FN N-terminal domains are involved in direct interactions of the proteins. (**A**) Interaction of full-length FN (100 µg/mL; coated on plate) with HSP90α domains or GST control (varying concentration in solution). Binding was detected using an anti-GST antibody. (**B**) Interaction of HSP90 full-length and M domain with FN fragments. (**C**) Interaction of HSP90M domain (100 µg/mL; coated on plate) with FN 30, 45, 70, and 120 kDa fragments (varying concentrations in solution). (**D**) Effect of inhibitors on the interaction between FN 30-kDa fragment and full-length HSP90α. Data represent averages (±SD, *n* = 3). Statistical analysis was conducted by two-way ANOVA and Bonferroni post-test, where * *p* < 0.05, ** *p* < 0.01, *** *p* < 0.001 and ns = not significant.

We previously reported the direct interaction of full-length fibronectin (FL-FN) with both full-length HSP90α and HSP90β. We first aimed to identify which domain of HSP90 binds FL-FN. We used recombinant GST-tagged HSP90α domains or GST as a control (Figure 1A), and commercially available FL-FN in a direct protein–protein binding assay. Only the GST-HSP90αM domain bound to FL-FN with greater affinity than the GST control. The N- and C-terminal domains of GST-HSP90α bound with similar or lower affinity compared to the GST control, suggesting that the observed interaction was attributable to GST (Figure 2A).

Having shown the association of GST-HSP90αM with FL-FN, we attempted to identify the region of FL-FN binding to HSP90αM. FN is made up of two identical 250-kDa subunits, which are interconnected by a pair of antiparallel disulfide linkages at the C-terminal end. FN is a modular protein, composed of repeating units of three types of domains, namely 12 FN type-I repeats, 2 FN type-II repeats, and 15 FN type-III repeats, each having a unique affinity and binding site based on cellular requirements (Figure 1B). Proteolytic treatment of full-length FN with cathepsin D gives rise to a 70-kDa N-terminal fragment (FN70, ^1–5^FNI^1–2^FNII^6–9^FNI) which is involved in FN assembly and can be cleaved by tryptic digest into two smaller fragments of 30 kDa (^1–5^FNI) and 45 kDa (^6^FNI^1–2^FNII^7–9^FNI) that have the ability to bind heparin and gelatin, respectively. The 120-kDa fragment (^1–11^FNIII) contains the integrin recognition site (RGD peptide) and the synergy site involved in cell binding required for unfolding and matrix assembly (Figure 1B).

Using the N-terminal fragments, we conducted a single point assay to identify the FN domain interacting with both full-length HSP90α and the M domain. The FN30 and FN70 fragments bound significantly more to the full-length HSP90α than the full-length FN. All of the FN domains and the full-length FN showed higher binding to the HSP90α domain alone than to the full-length protein, with the FN30 domain showing the highest binding (Figure 2B). Next, we conducted a dose-dependent analysis using the FN fragments and HSP90αM domain. In this analysis, FN30 had a dissociation constant (K_d_) value that was 3-fold lower than that of FN45 and 8-fold lower than that of FN70, suggesting a strong physical association (Figure 2C). FN45 also bound to HSP90αM with lower affinity than FN30, but with greater affinity than FN70. We did not observe any significant direct binding of FN120 to HSP90αM.

Next, we tested the effect of HSP90 modulators on the interaction with FN30. The HSP90 inhibitor 17-DMAG (N-terminal ATP competitive inhibitor) reduced, but did not significantly alter, the interaction, while novobiocin (C-terminal inhibitor) had no effect. Sodium molybdate (NaMb), which retains the HSP90 client complex in a pseudo ATP bound state, significantly reduced the interaction of HSP90α and FN30 equivalent to that of 17-DMAG (Figure 2D).

### 3.2. Colocalization of Fibronectin and HSP90α in Cells

**Figure 3:**
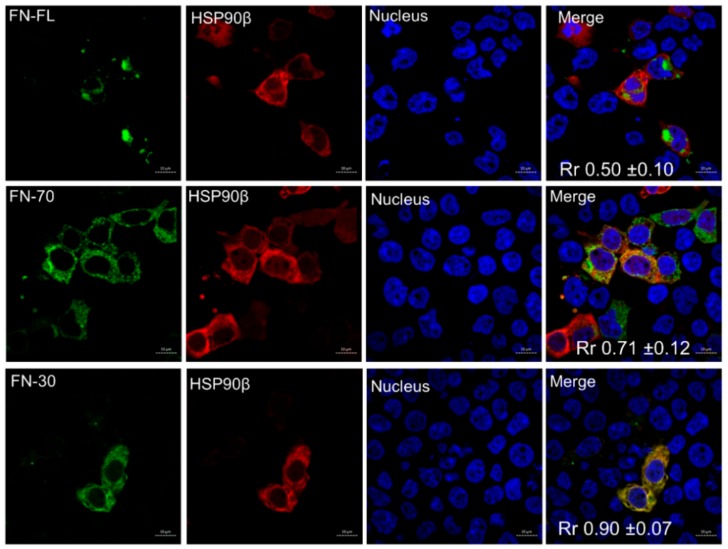
HSP90 and fibronectin fragments colocalize in cells. Colocalization analysis of mCherry-HSP90β (red) and full-length FN-YPET, FN30-EGFP, and FN70-EGFP (green) by confocal microscopy. Scale bar is 10 µm and data shown are representative of independent biological triplicate experiments. The Rr shows the average Pearson’s correlation coefficient (±SD, *n* ≥ 3) between HSP90 and FN signals.

Soluble FN is processed through the Golgi apparatus and the secretory pathway to the extracellular matrix (ECM); hence, the protein exists both as intracellular and extracellular forms. Therefore, we assessed the colocalization of HSP90α and FL-FN, FN30, and FN70 in cells. HEK293T cells were co-transfected with fluorescent fusion proteins of FN and HSP90β (Figure 3). mCherry-HSP90 colocalized in cells expressing all the FN proteins, with the FN30-EGFP fragment showing the highest degree of colocalization (Pearson’s correlation coefficient, Rr = 0.9 ± 0.07), followed by FN70-EGFP (Rr = 0.71 ± 0.12) and FN-YPET (yellow fluorescent protein variant) (Rr = 0.5 ± 0.10). Interestingly, despite the morphology of the staining, we did not observe any significant colocalization of FN-YPET with a marker of the Golgi apparatus, while FN30-EGFP, which interacted and colocalized more with HSP90 showed the highest degree of colocalization with the Golgi apparatus (Figure S1).

### 3.3. Analysis of FN and HSP90α Interaction by Biomolecular Fluorescence Complementation (BiFC) Assay

**Figure 4:**
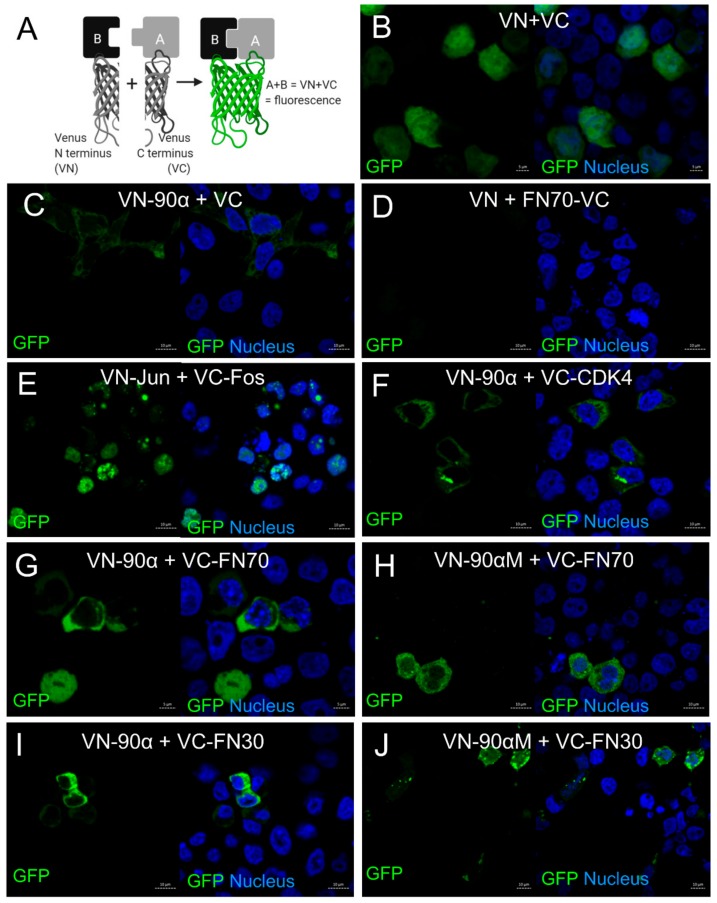
HSP90 and fibronectin interact directly in cells. Biomolecular complementation (BiFC) assay using split mVenus tagged FN and HSP90 proteins in HEK293T cells. (**A**) Schematic diagram of the BiFc method for measuring protein–protein interactions in cells. Images shown co-transfection of (**B**) empty vectors pBiFC-VN173 (VN) and pBiFC-VC155 (VC) (negative control), (**C**) pBiFC-VN173-HSP90α (VN-90α) and VC (negative control), (**D**) pBiFC-VC155-FN70 (FN70-VC) and VN (negative control), (**E**) pBiFc-VN173-bJun (VN-Jun) and pBiFC-VC155-delbFos (VC-Fos) (positive control), (**F**) VN-90α and pBiFc-VC155-CDK4, (**G**) VN-90α and VC-FN70, (**H**) pBiFc-VN173-90α M domain (VN-90αM) and VC-FN70, (**I**) VN-90α and VC-FN30, and (**J**) VN-90αM and VC-FN30. In all cases, endogenous fluorescent signal (GFP; green) was captured by confocal microscopy using the EGFP filter set. Scale bars are equivalent to 5 or 10 µm and data shown are representative of biological triplicate experiments.

Solid-phase binding assays provide details on direct in vitro interaction, but do not provide information on the interaction in cells. Colocalization suggests that proteins are located in similar cellular regions to permit interaction, but does not guarantee direct physical association in cells. We therefore wanted to investigate the direct binding in cells using the biomolecular fluorescence complementation (BiFC) assay [48] (Figure 4).

In this assay, potential interacting partners are fused to the N or C terminus of the mVenus protein (denoted VN or VC, respectively). The split mVenus fluorophore is able to form if the two fusion proteins interact within a specific distance of 10 nm or less [58] (Figure 4A). HEK293T cells were co-transfected with BiFC plasmids encoding FN fragments or HSP90α, or relevant controls (see Supplementary File for details) and the endogenous GFP signal was analyzed by confocal microscopy. To confirm that the BiFC assay was working, we co-transfected the empty backbone plasmids (encoding only VN or VC) (Figure 4B), or an empty backbone plasmid together with the corresponding plasmid encoding either VN-HSP90α or VC-FN as negative controls (Figure 4C,D). These controls either produced low GFP fluorescence that was diffuse in the cells or no GFP fluorescence. In contrast, the positive control with known interactors, VN-Fos and VC-Jun, produced a strong GFP signal in the nucleus as expected (Figure 4E). We further confirmed that the BiFC could be used to identify chaperone–client interactions by co-transfection of VN-HSP90α with VC-CKD4, a well-known HSP90 client protein (Figure 4F). Supporting the initial observation in solid-phase interaction assays, BiFC showed a similar trend of binding for HSP90α and FN fragments. We obtained a strong signal in BiFC from VC-FN70 and VC-FN30 with either VN-HSP90α or HSP90αM which was above the background signals (Figure 4G–J). The distribution of the GFP signal in the HSP90α and FN30/FN70 transfections was similar to that observed in colocalization analyses (Figure 3). Clear puncta were observed around the nucleus and near the cell membrane in cells transfected with VC-FN30 and VN-HSP90αM or HSP90α. The signal from the VC-FN70 and VN-HSP90αM or VN-HSP90α was weaker and more diffuse, thus supporting our initial finding of stronger interaction of HSP90 with FN30 than FN70. The intensity of the signal was not linked to expression, since all the proteins showed similar expression levels (Supplementary Figure S2).

### 3.4. Analysis of the Effect of FN Fragment Stability on HSP90 Interaction

HSP90 client proteins are thought to be metastable and the degree of instability is linked to the requirement for HSP90 interaction. Following the biochemical analysis of the FN and HSP90 interaction, we tested the stability of the FN fragments using the SYPRO Orange-based thermal shift assay (Table 1). In this assay, SYPRO orange fluorescence increases as it binds to hydrophobic regions which are exposed during a gradual heating of a protein solution. A lower T_m_ of unfolding indicates a less stable protein. FN30 had the lowest T_m_ of unfolding, followed by FL-FN, FN45, and FN70, the latter was surprisingly more stable than the full-length protein. The low T_m_ for FN30 suggested that it is the least stable and correlates with the strongest HSP90 interaction.

### 3.5. FN70 and HSP90 Interaction in the Presence of Heparin and Gelatin

**Figure 5:**
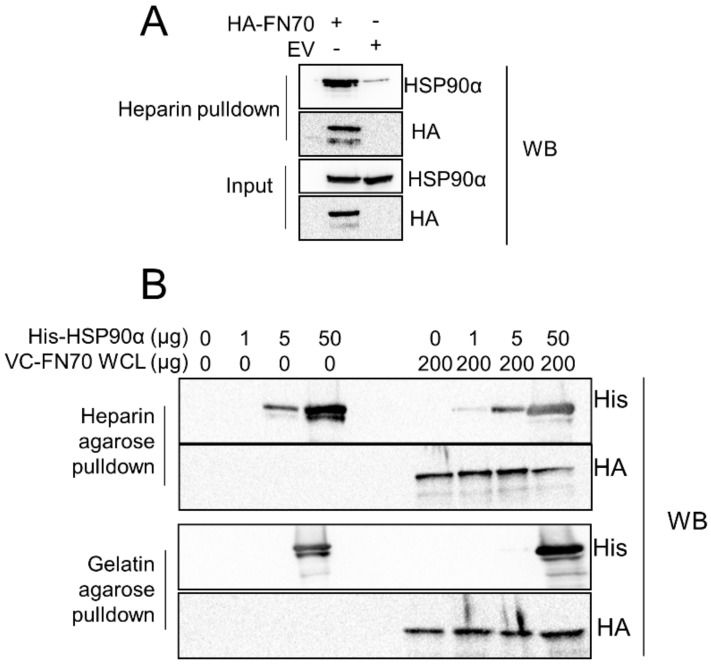
FN interaction with HSP90 and heparin is not mutually exclusive. (**A**) FN70 and HSP90 interaction in the presence of heparin and gelatin. (**B**) Heparin (upper panel) and gelatin (lower panel) agarose pull-down assay using whole cell lysate (WCL) from HEK293T cells transfected with VC-FN70 and exogenous recombinant purified His-HSP90α. Complexes were analyzed by western blot with an anti-HA antibody to detect VC-FN70 and an anti-His antibody to detect His-HSP90α. Data shown are representative of two independent replicates.

Our data from in vitro and BiFC analyses suggested that HSP90α could interact with FN70 and the two smaller FN30 and FN45 proteolytic fragments. We therefore attempted to refine the binding site for HSP90α in FN70 by using the ability of the FN30 and FN45 regions to selectively bind heparin and gelatin, respectively. Additionally, HSP90 has previously been shown to bind heparin [59], which allowed us to assess if HSP90 binding to heparin would be competitive with FN70. FN70-transfected cell lysate was added to heparin beads and the level of endogenous HSP90 in the heparin-bound protein complex was determined (Figure 5A). FN70-transfected cells were found to contain more endogenous HSP90α than non-transfected cells, suggesting that the binding of these proteins to heparin was not mutually exclusive. Next heparin-conjugated and gelatin-conjugated agarose beads were used to expand the binding analysis. We monitored the amount of recombinant HSP90 and FN70 from cell lysates that could be recovered from the heparin/gelatin bound beads. HSP90α bound to heparin agarose in the absence of FN70, but was increased in the heparin complexes in the presence of FN70 (Figure 5B, upper panel). Interestingly, at the highest amount (50 µg) of HSP90α, there was a slight, but consistent, reduction in FN70 binding. HSP90 bound to gelatin in the absence of FN70 only at the highest concentration, and the trend of binding did not change in the presence of FN70 (Figure 5B, lower panel). Taken together, these data suggested that despite binding the FN30 and FN45 domains, HSP90 does not disrupt interactions of FN70 with heparin or gelatin, indicating that the interaction sites do not overlap.

### 3.6. HSP90 and FN Interaction Involves Type-I FN Motifs

**Figure 6:**
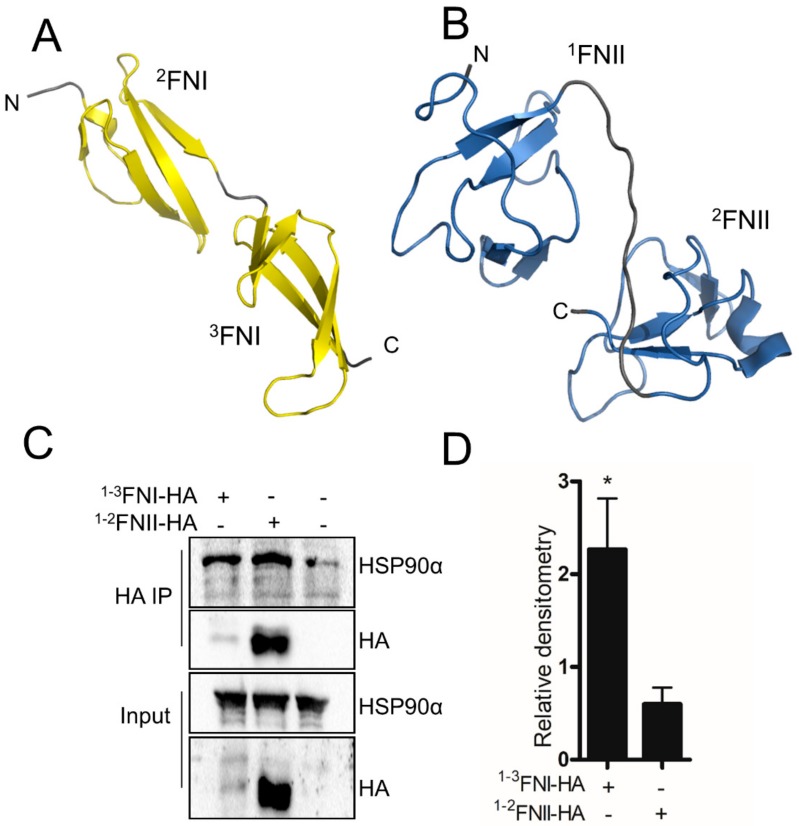
HSP90α interacts preferentially with type-I FN repeats. Three dimensional structures of (**A**) the second and third type-I FN repeat from the 30/70-kDa domains (PDB: 2CG6) and (**B**) the first and second type-II FN repeats from the 45/70-kDa domains (PDB: 3MQL). (**C**) Co-IP from HEK293T cells transfected with plasmids encoding the first to third FN type-I repeats (3X-FN-TI-HA) or the two type-II repeats (2X-FN-TII-HA). (**D**) Detection of interacting proteins by western blotting (*n* = 3). Statistical analysis was performed using two-way ANOVA, where * *p* < 0.05.

HSP90 is thought to mainly recognize unstable conformations rather than sequence motifs. Given the modular nature of the FN protein, we wondered if HSP90 would preferentially recognize a particular repeat type and if this would contribute to the interaction affinity. The FN30 fragment is composed solely of type-I FN motifs (^1–5^FNI) (Figure 6A), whereas the FN45 has both type-I and type-II motifs (^6^FNI^1–2^FNII^7–9^FNI) (Figure 6B). Both repeats contain disulfide-bonded cysteines and are comprised mainly of β-sheets connected by loop regions (Figure 6). We generated expression plasmids containing only the type-I (^1–3^FNI) or type-II FN repeats (^1–2^FNII) and performed HA co-immunoprecipitation (Co-IP) to compare the binding affinity of HSP90. We observed that more HSP90 was isolated in complex with ^1–3^FNI compared to ^1-2^FNII, despite substantially higher expression of ^1–2^FNII (Figure 6C,D).

### 3.7. Interaction of FN with Conformationally Restricted HSP90 Mutants

**Figure 7:**
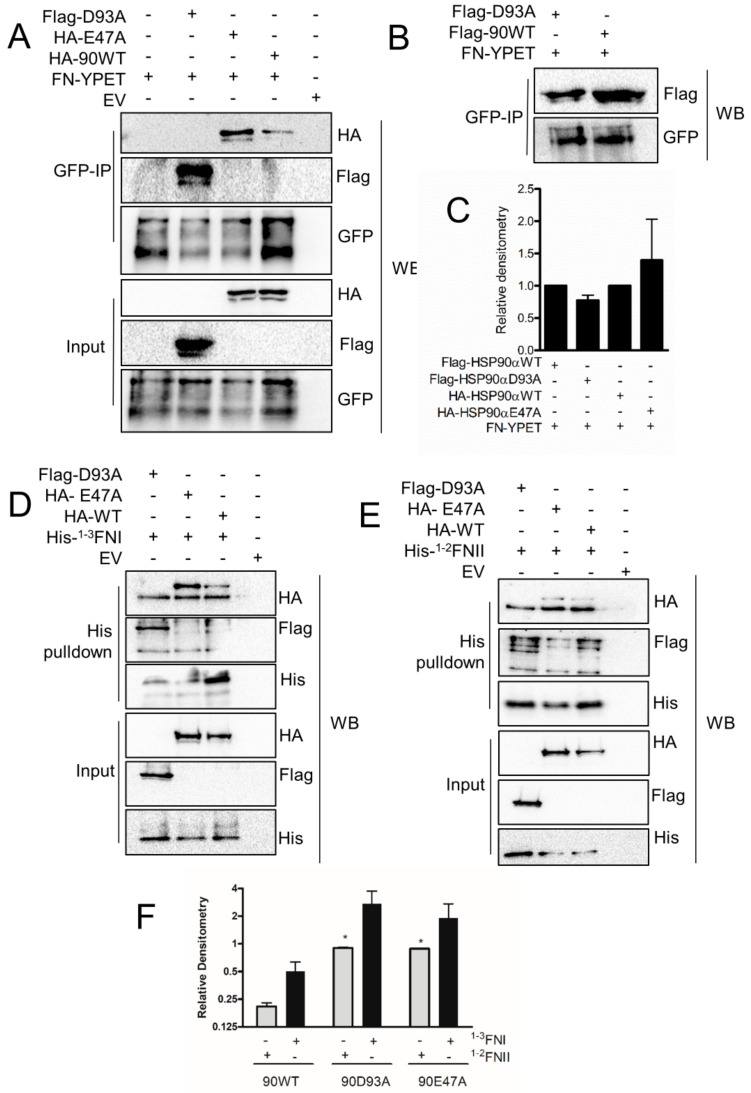
FN type-I and type-II repeats interact preferentially with conformationally restricted HSP90 mutants. Co-IP/pull-down linked to western blot analysis from HEK293T cell lysates transfected with full-length FN-YPET and (**A**) pcDNA-HA-HSP90α (HA-90WT; wild-type HSP90α), pcDNA-HA-HSP90 E47A (HA-E47A; closed conformation mutant), or pcDNA-Flag-HSP90 D93A (FLAG-D93A; HSP90α open conformation mutant) or (**B**) pcDNA-Flag-HSP90 (Flag-90WT; HSP90α wild-type) or FLAG-D93A. (**C**) Average relative densitometry normalized to wild-type HSP90 (±SD, *n* = 2). Co-IP/pull-down linked to western blot analysis from HEK293T cell lysates transfected with either (**D**) pcDNA-His-FN type-I repeats (His-FN-TI) or (**E**) pcDNA-His-FN type II repeats (His-FN-TI), and HA-90WT, HA-E47A, or FLAG-D93A. (**F**) Average relative densitometry (±SD, *n* = 2). EV indicates empty vector control. Statistical analysis was performed using two-way ANOVA, where * *p* < 0.05.

The ATP-coupled HSP90 chaperone machinery works through sophisticated conformational changes, where it adopts a ‘closed’ or ‘open’ state in reference to the dimerization of the N-terminal domains. These different HSP90 conformations are known to interact differently with some client proteins and co-chaperones. We therefore performed immunoprecipitation from HEK293T cells transiently co-transfected with wild-type HSP90 or the open (D93A) or closed (E47A) mutants and the full-length expression plasmid FN-YPET. FN-YPET interacted with both with the ‘open’ and ‘closed’ HSP90 conformation; however, the interaction with the open conformation (D93A) was lower (Figure 7A–C). In contrast, the ^1–3^FNI and ^1–2^FNII FN motifs bound more strongly to both the open and closed HSP90 conformations compared to the wild-type HSP90, with ^1-3^FNI consistently binding more to all HSP90 variants than ^1–2^FNII (Figure 7D–F).

### 3.8. Phosphorylation of HSP90-Y313 does not Affect. HSP90–FN Interaction

**Figure 8:**
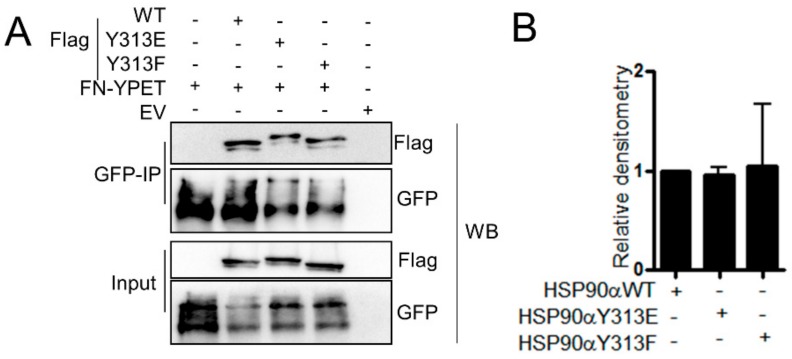
Mutation of HSP90-Y313 does not alter FN interaction. (**A**) Co-IP from HEK293T cells transfected with (**A**) pcDNA-Flag-90WT (WT), pcDNA-Flag-HSP90Y313E (Flag-Y313E; phosphomimetic), or pcDNA-Flag-HSP90Y313F (Flag-Y313F; phosphomutant) and (**B**) associated densitometry (±SD, *n* = 3) relative to wild-type HSP90. EV indicates empty vector control.

Phosphorylation has an important role in regulating the HSP90 chaperone cycle through regulation of ATPase activity, co-chaperone association, and client recognition. The activation of ATP hydrolysis by the co-chaperone Aha1 (activator of HSP90 ATPase 1) enhances the release of HSP90 client proteins from the complex. Aha1 recruitment and, by extension, client release are enhanced by Y313 phosphorylation in the middle domain of HSP90α [52]. Subsequently, we wanted to test if this phosphorylation had any significant impact on HSP90–FN interaction. An immunoprecipitation assay was performed after transfection of a phosphomimetic tyrosine mutant (Y313E), a phosphomutant (Y313F), or wild-type HSP90α. We did not see any significant changes with HSP90A-Y313E or HSP90A-Y313F compared to wild-type HSP90 (Figure 8A,B).

### 3.9. Extracellular HSP90 Increases the FN Matrix In Vitro

**Figure 9:**
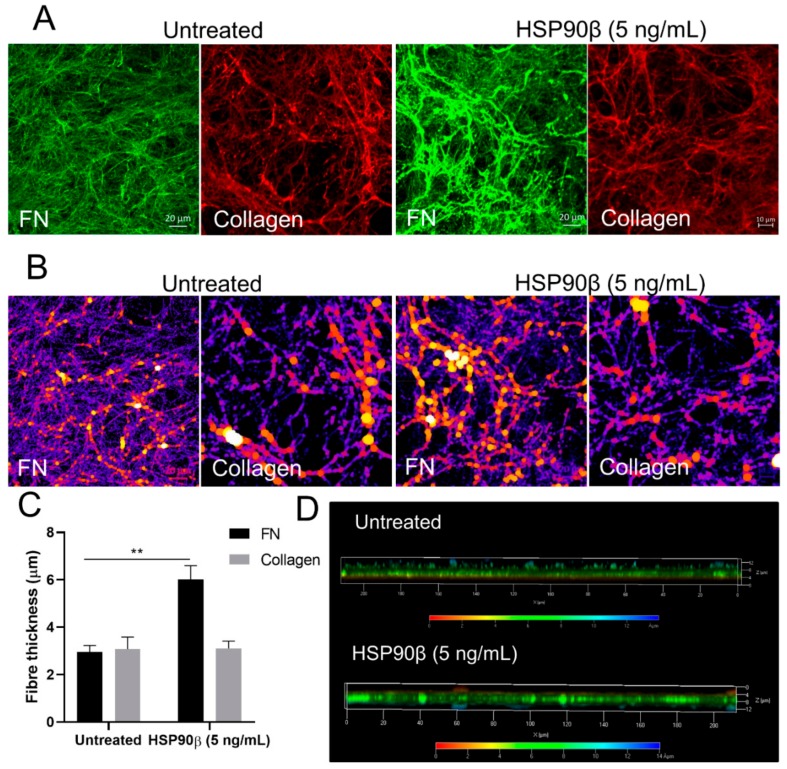
HSP90 treatment increases the thickness of FN fibrils in the extracellular matrix of Hs578T cell-derived matrices. (**A**) Cell-derived matrices (CDMs) stained for FN (green) or collagen (red). Scale bars represent 20 μm. (**B**) The BoneJ plugin [60] in ImageJ was used to calculate fiber thickness, where pseudocolored spheres that fit along fibers represent intensity maps of fiber thickness. (**C**) Quantification of fiber thickness represented as mean thickness (±SD) taken from triplicate independent images. Statistical significance was determined by unpaired Student’s t-test (** *p* < 0.01). (**D**) Depth coding of representative 3-dimensional z-stack projections of CDM. Scale bar shown below is color-coded to indicate the depths of matrices in µm.

We have shown previously that HSP90β maintains the stability of the FN matrix, since treatment of cells with HSP90 inhibitors induced internalization and loss of extracellular FN via a mechanism requiring LRP1/CD91 [32,33]. Our current data showed that HSP90 interacts with FN via N-terminal regions, including the 70-kDa assembly fragment. To assess the effect of extracellular HSP90 on the FN matrix, we generated cell-derived matrices (CDM) from Hs578T breast cancer cells, which endogenously produce and assemble high levels of FN matrix [32]. Cells were allowed to develop a matrix over 5 days, and then left untreated or treated for 24 h with exogenous endotoxin-free HSP90β. After cell removal, the remaining FN cell-free matrix was visualized by immunofluorescence and confocal microscopy (Figure 9A). Matrices from HSP90β-treated cells had significantly thicker FN fibrils, whereas untreated matrices had uniformly thin, long fibrils (Figure 9B,C). In addition, FN matrices in HSP90-treated cells had increased depth compared to untreated matrices (Figure 9D). This phenotype was exclusive to FN, as the same trend was not seen in collagen fibrils (Figure 9). This suggested that HSP90 may promote FN fibril assembly.

**Figure 10:**
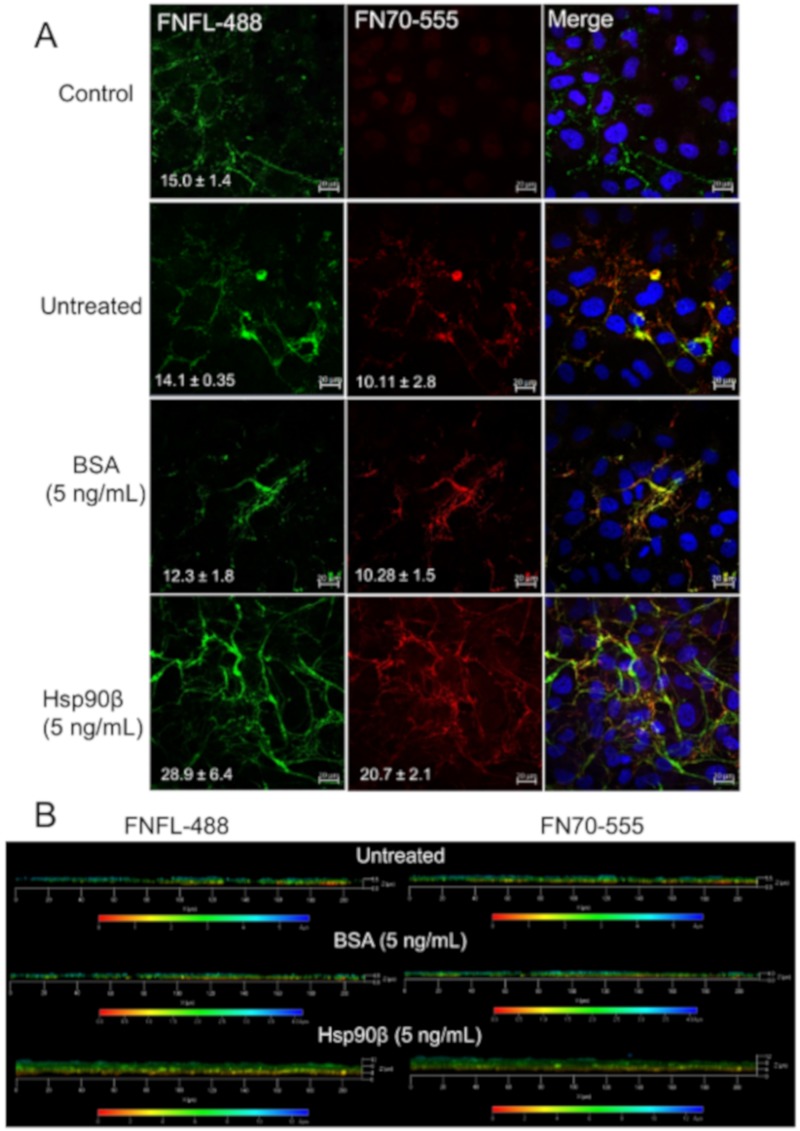
HSP90 treatment increases the incorporation of exogenous FN into fibrils. (**A**) Incorporation of exogenous fluorescently labeled FN into the matrix of Hs578T breast carcinoma cells. Images were captured on a Zeiss LSM 780 laser scanning confocal microscope and analyzed using the Zen Blue software (Zeiss, Germany). Data are representative of triplicate independent studies. Scale bars represent 10 μm. (**B**) Depth coding of representative 3-dimensional z-stack projections of matrices. Scale bar shown below is color-coded to indicate the depths of matrices in µm.

Fibril changes in the CDM model could be influenced by changes in FN expression or secretion, and HSP90 has been associated with FN trafficking in LNCaP prostate carcinoma cells [37]. Consequently, we also assessed the ability of fluorescently labeled exogenous FN to be incorporated directly into ECM fibrils (Figure 10). Hs578T cells were incubated with exogenous Dylight-488 fluorescent dye-conjugated full-length human FN (FL-FN-488) for 24 h, followed by the addition of exogenous Dylight-555 red fluorescent dye-conjugated human FN70 fragment (FN70-555) with or without extracellular HSP90β for 1 h. The resulting matrices were analyzed by confocal microscopy. We observed increased incorporation of both full-length FN (green) and FN70 (red) into matrix fibrils in HSP90-treated cells compared to untreated cells (Figure 10A) and matrices showing increased depth (Figure 10B). The BSA control treatment resembled the untreated control, suggesting that the increase was not due to the additional protein, but rather was specific to the addition of HSP90β. Together, these data suggested that exogenous HSP90β promotes incorporation of soluble FN and FN70 into fibrils, which may be through the ability to interact with the FN70 fragment.

### 3.10. C-terminal, but not N-terminal, HSP90 Inhibitors Reduce Total FN Levels In Vitro

**Figure 11:**
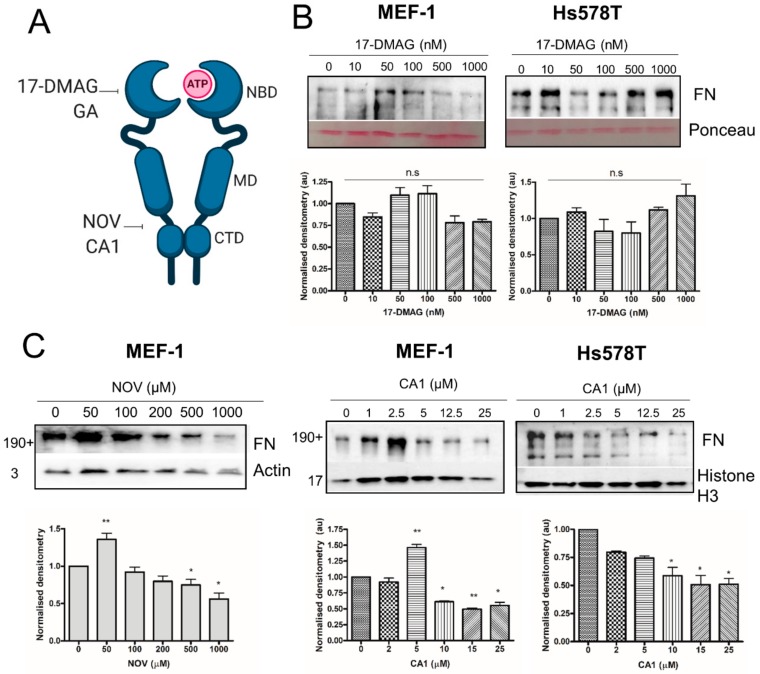
C-terminal HSP90 inhibitors reduce total FN levels in fibroblasts and breast cancer cells. (**A**) Schematic diagram showing the HSP90 domains targeted by inhibitors (NBD: N-terminal nucleotide-binding domain; MD: middle domain; CTD: C-terminal domain). Treatment of MEF-1 and Hs578T breast carcinoma cell lines with increasing concentrations of the (**B**) N-terminal HSP90 inhibitor, 17-dimethylamino-ethylamino-17-demethoxydeldanamycin (17-DMAG) or (**C**) C-terminal HSP90 inhibitors, novobiocin (NOV) or coumermycin A1 (CA1). Levels of FN in whole cell lysates were determined by western blot and average densitometry (±SD, *n* = 3) using ImageJ. Images are representative of triplicate experiments. Statistical significance was determined using a one-way ANOVA and Bonferroni post-test in GraphPad Prism 4 (* *p* < 0.05, ** *p* < 0.01, ns = not significant).

To support our observations of increased FN matrix in HSP90β-treated cells, we tested the effect of non-toxic concentrations of HSP90 inhibitors on FN levels by western blot in both the Hs578T breast cancer cell line and the MEF-1 fibroblast line (given the core role of fibroblasts in ECM production) (Figure 11). HSP90 inhibitors that bind to the N-terminal (e.g., geldanamycin and 17-dimethylamino-ethylamino-17-demethoxydeldanamycin (17-DMAG)) and C-terminal (e.g., novobiocin (NOV) and coumermycin (CA1)) domains of the protein have been identified (Figure 11A). Consistent with our previous studies, N-terminal inhibition of HSP90 with 17-DMAG did not significantly alter the levels of total FN in MEF-1 or Hs578T cells (Figure 11B). C-terminal inhibition of HSP90 with CA1 in Hs578T cells resulted in a significant dose-dependent decrease in total FN levels. Interestingly, in the MEF-1 cell line, low doses of CA1 or NOV led to a significant increase at low concentrations, followed by a dose-dependent decrease in total FN levels (Figure 11C). 

### 3.11. C-Terminal Hsp90 Inhibitors Alter Cell Migration In Vitro

**Figure 12:**
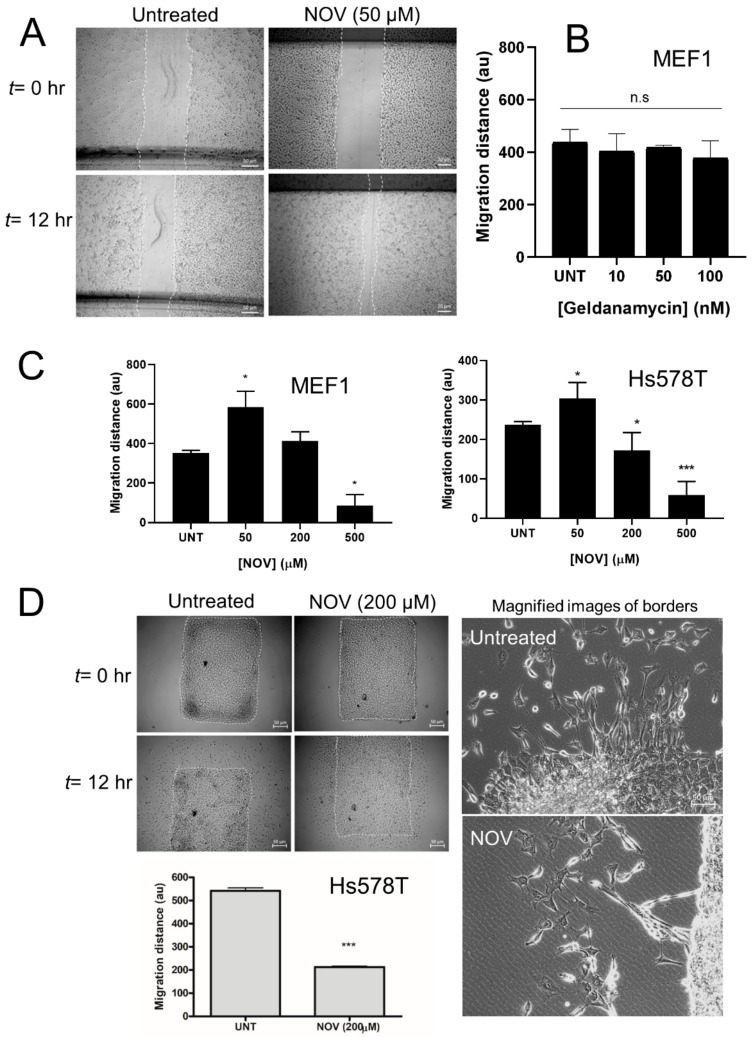
Effect of HSP90 on cell migration of fibroblasts and breast cancer cells. (**A**) Representative image of linear wounds created in MEF-1 cells during the scratch assay with NOV treatment. White lines indicate cell migration borders. Quantitation of migration in scratch assay for (**B**) MEF-1 fibroblast line treated with the N-terminal HSP90 inhibitor, geldanamycin and (**C**) MEF-1 fibroblast line or Hs578T breast carcinoma treated with the C-terminal HSP90 inhibitor, novobiocin (NOV). (**D**) Migration away from a monolayer culture was measured in the Hs578T cell line. White lines indicate cell migration borders. Average distances (±SD) for migration outward from the cell border at *t* = 12 h were measured using ImageJ. Statistical analysis was performed using unpaired two-tailed t-tests, where * *p* < 0.05, *** *p* < 0.001, ns = not significant. Images are representative of averages (±SD) of triplicate independent experiments.

We next analyzed if treatments which altered FN levels or morphology would result in changes in cell migration in the MEF-1 and Hs578T cell lines using the scratch assay (Figure 12A). The N-terminal HSP90 inhibitor, geldanamycin, did not affect cell migration in the MEF-1 cell line, which was consistent with no changes observed in the FN levels (Figure 12B). NOV treatment led to an increase in cell migration at low concentrations, followed by a dose-dependent reduction in migration in both cell lines. This cell migration response was consistent with the trend in total FN levels in the MEF-1 cell line (Figure 12C). We also tested the ability of Hs578T cells that had been pre-treated with NOV to migrate from the monolayer (Figure 12D). NOV treatment significantly reduced the migration of Hs578T cells compared to the control. The control Hs578T migrated collectively from the monolayer, while the NOV-treated cells migrated as single cells (Figure 12D).

## 4. Discussion

FN is a major constituent of the ECM [61,62,63,64]. It is synthesized as a soluble protein and assembled into an insoluble fibrillar matrix outside of cells [44,65,66]. The FN matrix is dynamic and the extent of the matrix is governed by balance between fibrillogenesis, which converts soluble FN into insoluble ECM, and turnover, a process which requires proteolytic cleavage, internalization, and degradation of the FN ECM [67,68]. The features of this matrix regulate cellular biology and are often perturbed in diseases, including cancer and fibrosis [69,70,71,72]. Therefore, the factors which regulate matrix assembly and turnover are important in understanding the diseases.

N-terminal HSP90 inhibition was associated with reduction in FN secretion in prostate cancer [37] and levels of HSP90 in the circulation were correlated with disease severity in patients with idiopathic pulmonary fibrosis (IPF) [38]. We previously described a link between FN and HSP90, demonstrating that the two proteins interacted directly in vitro and could be isolated as a complex from cells [30]. HSP90 inhibition led to either a loss in extracellular FN (which was dependent on LRP1 expression) or an increase in FN expression in vitro, depending on whether C-terminal or N-terminal targeted inhibitors were used, respectively [30,31,73]. Here, we extend this analysis to identify the molecular basis for the FN–HSP90 interaction, which provides potential for mechanistic insights into the role of HSP90 in FN matrix biology. HSP90 directly interacted with FN via the M domain and in both the open and closed conformations. The HSP90 inhibitors, sodium molybdate and 17-DMAG, reduced FN–HSP90 association, while phosphorylation of HSP90 at Y313, which promotes recruitment of Aha1 to the M domain of HSP90 [50,74], did not influence FN binding indicating that the Aha1- and FN-binding sites on the M domain are distinct. HSP90 preferentially recognized the type-I FN motif and was able to interact with the N-terminal proteolytic FN fragments (FN70, FN45, and FN70) in vitro and in HEK293T cells, but did not associate with the FN120 cell-binding fragment. The FN30 fragment was the least stable and bound HSP90 with the greatest affinity. HSP90 binding did not interfere with the ability of the FN70 domain to interact with heparin or gelatin, and promoted the incorporation of both full-length FN and FN70 into the assembled matrix when added exogenously to Hs578T cells in culture.

HSP90 binds to a wide set of substrate proteins known as ‘clients’, and confers stability to and promotes proper folding of these proteins [1,7,10,75]. In addition to conformational regulation, HSP90 has been shown to suppress aggregation and promote protein solubility, activities which in vitro appear to be ATP-independent [76,77]. There are two major deciding factors that define client proteins, namely, dependence on HSP90 for stability and direct association with HSP90, both of which have been demonstrated for FN [30]. The ability of HSP90 to undergo conformational changes is central to its function as a chaperone. These conformational changes are driven primarily by ATP association, and can be mimicked by specific mutants which trap the chaperone in specific states. In the case of some client proteins like HSF1 [78] and ERBB2 [51], binding to the closed conformation is preferred. FN, consistent with other client proteins like HIF1α, MET kinase, and RHOBTB2 bound both the open and closed conformations of HSP90 [51,79]. In addition, both 17-DMAG and sodium molybdate inhibit the progression of HSP90 through the chaperone cycle by different mechanisms, and both reduced FN30 association with HSP90, although only the effect of molybdate was significant. The fact that 17-DMAG disrupted the interaction of HSP90 and FN may be expected from other client protein responses [80,81,82]. Sodium molybdate, in contrast, retains HSP90 in an ATP bound state [83], which has been shown to promote complex formation and stabilize other client interactions [84,85]. These data differentiate FN from other client proteins like steroid receptors and suggest that ATP binding may destabilize the FN30–HSP90 interaction. This is interesting since the requirement for ATP for extracellular HSP90 functions remains contentious.

HSP90 clients are structurally and functionally diverse and comprise partially folded, disordered, and folded proteins [6,86,87,88]. The binding site is not confined to a specific region, but rather involves several lower affinity binding points, which together form a stable transient state [28]. Client–HSP90 interactions mainly possess binding affinities in the low micromolar range, for example, HSP90–Tau interaction in the ATP bound state has a K_d_ of 4.8 µM [89], while the K_d_ of the ATP-bound yeast HSP90 to the ligand-binding domain of glucocorticoid receptor (GR-LBDm) is 0.8 µM [90]. The affinity of FN70 for HSP90 was within this range (K_d =_ ~0.5 µM), while the HSP90–FN30 interaction was significantly stronger (K_d =_ ~0.07 µM). This high affinity does not seem consistent with the transient, lower affinity interactions of HSP90 with clients, despite the fact that the HSP90 M domain, to which FN bound, is regarded as the major client-binding domain [13,91]. The HSP90 M domain plays a critical role in client selection and binding [13] and modulates substrate activation. The molecular determinants for HSP90 recognition across such a diverse group of client proteins remain poorly defined. Most published reports suggest that HSP90 preferentially binds to metastable protein conformations [92,93] rather than interacting with linear sequence motifs. From the analysis of complexes of Tau, cyclin dependent kinase 4 (CDK4), and GR with HSP90 [28,94,95,96], the chaperone binding regions in client proteins tend to extend across a larger surface, be less hydrophobic than those recognized by HSP70, and contain stretches of positively charged residues [89]. The FN30 fragment, which bound HSP90 with the highest affinity, is consistent with interactions involving aggregation-prone proteins like Tau and transthyretin [28,86], in that FN30 has a basic pI (8.2–8.6) compared to the full-length protein (pI: 5.5–6.0), making it positively charged at physiological pH. FN30 is comprised solely of repeats of type-I FN motifs (^1–5^FNI) [97], which form a disulfide-bonded, triple-stranded, β-sheet connected by loop regions. An increase in β-sheets has been linked to increased aggregation in a number of proteins [86] and two adjacent unfolded β-sheets from CDK4 are captured in the hydrophobic core of HSP90 [89]. In addition, loop regions in kinase clients are important for HSP90 recognition [98]. Interestingly, FN30 has several tandem β-sheets linked by loops that may account for its instability and recognition by HSP90 during the chaperone cycle [28,86]. The preferential binding of HSP90 to the type-I FN motif over the type-II motif might appear to explain the higher affinity binding for the FN30 (^1–5^FNI) fragment, as well as interaction with the FN45 fragment (^6^FNI^1–2^FNII^7–9^FNI) and lack of interaction with the FN120 cell-binding fragment (which is comprised solely of type-III FN repeats). The recognition of the type-I motif is not sufficient to explain the binding of FN to HSP90, since the full-length FN and FN70 contain more type-I repeats than the FN30/FN45 fragments and yet bind with lower affinity. This suggests that other factors, notably, the thermal stability of FN fragments may control the binding affinity for HSP90, as it does for other clients [29]. HSP90 may bind regions in clients important for both aggregation and folding [89]. Previous reports suggest that the FN30 fragment has a disordered structure [42]. Intrinsically disordered proteins or domains are those that lack a fixed conformation in the native state and may rather sample or adopt a series of different conformational states depending on conditions and these are enriched in the ECM [99]. The fact that HSP90 binds the full-length and FN70 proteins with a lower affinity despite the fact that both of these regions encompass the FN30 fragment suggests that stability may be the main driver of recognition and that the disordered motifs of FN30 are somehow different or stabilized in the full-length FN or FN70. Indeed, it is not known if the FN30 fragment is disordered in the context of full-length FN [42].

While FN30 is the least stable of the FN fragments tested, it is substantially more stable that other aggregation-prone HSP90 clients like Tau. The formation of insoluble FN is an important physiological feature of the protein, which distinguishes it from toxic aggregates of proteins like Tau. As such, the conversion of soluble FN to insoluble is a tightly regulated process, which is still not fully understood in molecular detail. The addition of exogenous HSP90 increased the extracellular FN matrix, resulting in thicker fibrils and a more three-dimensional matrix and promoting the assembly of both full-length FN and FN70 into matrices. Consistent with this and our previous studies [31,73], C-terminal HSP90 inhibitors, but not N-terminal HSP90 inhibitors, altered both FN levels and cell migration in breast cancer and fibroblast lines. The change in FN levels was consistent with the migratory phenotype in that increased levels of FN at low inhibitor concentrations led to increased migration, while cell migration was inhibited at higher levels and associated with reduced total FN. The regulation of FN levels and morphology may therefore represent one mechanism by which extracellular HSP90 regulates migration [21]. HSP90 did not influence collagen morphology, which is consistent with the fact that HSP90 does not affect FN interaction with collagen and that collagen is not required for FN matrix formation. Interestingly, while the integrin-binding regions of FN are found in the non-HSP90-binding FN120 fragment, the N-terminal regions of FN which bind HSP90 are required for FN matrix assembly. FN70 binds currently undefined cell surface receptors to promote FN assembly [100], although excess FN70 also blocked FN assembly. Of particular interest to this study is that FN lacking the first five type-I motifs (^1–5^FNI, encompassing the HSP90-interacting FN30 fragment) was unable to form fibrils [101,102,103]. The deletion of ^1-5^FNI fragments would potentially remove the major high affinity HSP90-binding site in FN. These data could therefore also be interpreted as highlighting a requirement for HSP90 interaction with FN for fibril formation, although this would still need to be experimentally confirmed; however, what is suggested is that the region and motifs in FN that interact preferentially with HSP90 are required for fibrillogenesis, providing some insight into one possible mechanism by which exogenous HSP90 regulates FN matrix assembly and supporting the modulation of HSP90 activity as a possible therapeutic approach for disorders in which FN dysregulation is a hallmark [30,31,37,38].

## Figures and Tables

**Table 1:** Analysis of stability of FN fragments by thermal shift assay.

Average T_m_ of Unfolding ± SD (°C) (*n* = 3)			
FN30	FN45	FN70	FL-FN
74.9 ± 1.7	77.6 ± 2.7	80.0 ± 4.5	76.4 ± 1.2

## Supplementary Materials

The following are available online at https://www.mdpi.com/2073-4409/9/2/272/s1, Figure S1: Colocalization of FN fragments with the Golgi apparatus, Figure S2: Confirmation of expression of proteins from BiFC plasmids; Table S1: Primer sequences used to generate expression plasmids used in this study, Table S2: PCR parameters used in this study, Table S3: Key resources used in this study and supplementary methods for protein expression and purification.
