# Supplementary material for: HSP90 Interacts with the Fibronectin N-terminal Domains and Increases Matrix Formation
Source: Cells. 2020 Jan 22;9(2):272. doi: 10.3390/cells9020272 (PMC7072298; doi:10.3390/cells9020272)
Supplement: Supplementary file 1 [file cells-09-00272-s001.pdf]

cells MDPI

Article

Analysis of the interaction between Hsp90 and the extracellular matrix protein, fibronectin

Abir Chakraborty [1], Natasha Marie-Eraine Boel [1] and Adrienne Lesley Edkins [1,2,*]

[1] Biomedical Biotechnology Research Unit, Department of Biochemistry and Microbiology, Rhodes University, Grahamstown
[2] Centre for Chemico- and Biomedicinal Research, Rhodes University, Grahamstown, 6140, South Africa

* Correspondence: a.edkins@ru.ac.za

Table S1: Primer sequences used to generate expression plasmids used in this study

Amplicon		Sequence of the primer (Forward=F and Reverse=R)
VC-FN30	F	AAGGGCCCGCCACCATGGGCATCCTTCCCAG
	R	AAAGGGCCCAACATCGGTGAAGGGGCCAGATCCG
VC-FN70	F	AAGGGCCCGCCACCATGGGCATCCTTCCCAG
	R	AAGGGCCCGCCACCATGGGCATCCTTCCCAG
VN-HSP90α-M	F	AAAAGATCTAGACAAGAAGAAGAAGAAGAAGATTAAGGAAAAGTACATCG
	R	GTCGACTTGAGCTTTCATGATTCTCTCCATGTTTGC
VN-HSP90α	F	AAAGTCGACCACCACCACCACCACCACATGCCGG
	R	AAAGTCGACATCAACCTCTTCCATACGGCTGGTATCGTCATCACCTTC
VC-CDK4	F	GAATTCAAATGGCTACCTCTCGATATGAGC
	R	GTCGACAACTCCGGATTACCTTCATCCTTATGTAG
FN30-EGFP	F	GGATCCGCCACCATGCTTAGGGGTCCGGGGC
	R	GAATTCAACATCGGTGAAGGGGCCAGATCC
FN70-EGFP	F	GGATCCGCCACCATGCTTAGGGGTCCGGGGC
	R	AAGAATTCGGTCTGTAAAGGTTGGCAATGCC

Table S2: PCR parameters used in this study

Name of the coding region	PCR parameter
VC-FN30, FN30 ,VN-HSP90αM and CDK4	98ºC/30s; (98ºC/30s, 58ºC/25s, 72ºC/50s) x 30 cycles; 72ºC/10 mins.
VC-FN70, FN70	98ºC/30s; (98ºC/30s , 58ºC/25s, 72ºC/60s) x 30 cycles; 72ºC/ 10 mins.
HSP90α	98ºC/30s: (98ºC/30s, 58ºC/25s, 72ºC/2.5 mins) x 30 cycles; 72ºC/ 10 mins.

Table S3: Key resources used in this study

Key resources	Source
pGEX-4T-1-GST-HSP90M	[1]
pGEX-4T-1-GST-HSP90N	[1]
pGEX-4T-1-GST-HSP90C	[1]
pGEX-4T-3-GST	[2]
pcDNA-Flag-HSP90αD93A	[3]
pcDNA-Flag-HSP90αWT	[4]
pcDNA-Flag-HSP90α-Y313E	[4]
pcDNA-Flag-HSP90α-Y313F	[4]
pHLSec2-FN-YPET	[5]
pcDNA-HA-HSP90αE47A	[6]
pcDNA-HA-HSP90αWT	[4]
pBiFC-VC155	[7]
pBiFC-VN173	[7]
pBiFC-VC155-CDK4	This study
pBiFC-VC155-del-bFos	[7]
pBiFC-VC155-FN30	This study
pBiFC-VC155-FN70	This study
pBiFC-VN173-HSP90α	This study
pBiFC-VN173-HSP90α-M	This study
pBiFC-VN173-bJun	[7]
pcDNA3-eGFP-FN30	This study
pcDNA3-eGFP-FN70	This study
pcDNA3-FN-3X-Type-I-HA-His	This study
pcDNA3-FN-2X-Type-II-HA-His	This study
mCherry-HSP90β	[8]
Anti GFP antibody	SC8334 (1:2000 dilution)
Anti HA antibody	Ab9110 (1:10000 dilution)
Anti Flag antibody	Ab49763 (1:10000 dilution)
Anti HSP90α antibody	ADI-SPA-840 (1:10000 dilution)
Anti His antibody	SC51946 (1:1000 dilution)
HRP-anti mouse secondary antibody	A16011 (1:5000 dilution)
HRP-anti rabbit secondary antibody	A16023 (1:10000 dilution)
HRP-anti rat secondary antibody	A18739 (1:5000) dilution
Anti GFP antibody (magnetic beads)	Ab193983
*Bam*HI, *Eco*RI,	NEB-R3136S, R3101S
T4 DNA Ligase	NEB-M0202S
*Apa*I, *Sal*I, *Bgl*I	NEB-R0114S,R-3138S,R-0144S
Protino glutathione agarose 4B	745500.10
Ni-NTA agarose beads	Qiagen 30210
pGEM-T and pGEM-T easy	Promega-A3600, A1360

Purification of recombinant proteins

For purification, GST-HSP90αM, GST-HSP90αN, GST-HSP90αC and GST were purified from induced *E.coli* XL1 Blue lysates according to the batch purification protocol in the Protino® Glutathione agarose 4B user manual. Protein expression was induced at OD600 0.6 with 1 mM IPTG for 4 hours. Cells were harvested at 6000 *xg* for 15 minutes at 4 °C. Cell pellets were resuspended in wash/equilibration buffer (50 mM Tris, pH 8.0, 150 mM NaCl) with Lysozyme (1 mg/ml), and PMSF (1 mM), and kept on ice for 20 minutes before incubating at -80°C overnight. Cell lysates were sonicated and centrifuged at 10000×*g* for 30 minutes at 4 °C. Supernatants were loaded into the

equilibrated Protino GSH Agarose beads and allowed to bind with the beads for 30-45 minutes at room temperature on rotating mixer. The beads were washed with wash buffer and proteins were eluted with GSH wash buffer containing 10 mM reduced glutathione. Purification was verified by SDS-PAGE.

His tagged HSP90α was expressed and purified from *E.coli* BL21(DE3) using codon optimized HSP90α sequence in the plasmid pET16b synthesized by Genscript (Hong Kong). At an OD600 of 0.6, the culture was treated with 0.5 mM IPTG to initiate protein expresion for 20 hours at 25 °C. Bacterial cells were harvested at 6000x*g* for 15 minutes at 4°C and the cell pellet was resuspended in His wash/lysis buffer (10 mM Tris pH 7.5, 300 mM NaCl and 50 mM imidazole) with lysozyme (1 mg/ml), and PMSF (1 mM). The cell lysate was fractionated at 16000x*g* for 30 mins at 4° C, and the supernatant loaded onto Ni^{+2}-charged sepharose beads followed by incubation overnight at 4°C on a rocker. Washing was performed with cold wash buffer containing 50-100 mM imidazole, and proteins eluted with cold elution buffer (10 mM Tris-HCl pH7.5, 300mM NaCl, 300 mM imidazole). All purified proteins (GST-tagged and His-tagged) were passed through a clean and concentration column with the appropriate MWCO filter prior to further analysis. Desalting was performed using Zeba spin columns as required.

References

1. Fontana, J.; Fulton, D.; Chen, Y.; Fairchild, T.A.; McCabe, T.J.; Fujita, N.; Tsuruo, T.; Sessa, W.C. Domain mapping studies reveal that the M domain of hsp90 serves as a molecular scaffold to regulate Akt-dependent phosphorylation of endothelial nitric oxide synthase and NO release. *Circulation Research* **2002**, *90*, 866–873.
2. Hunter, M.C.; O'Hagan, K.L.; Kenyon, A.; Dhanani, K.C.H.; Prinsloo, E.; Edkins, A.L. Hsp90 binds directly to fibronectin (FN) and inhibition reduces the extracellular fibronectin matrix in breast cancer cells. *PLoS ONE* **2014**, *9*.
3. Woodford, M.R.; Sager, R.A.; Marris, E.; Dunn, D.M.; Blanden, A.R.; Murphy, R.L.; Rensing, N.; Shapiro, O.; Panaretou, B.; Prodromou, C.; et al. Tumor suppressor Tsc1 is a new Hsp90 co-chaperone that facilitates folding of kinase and non-kinase clients. *The EMBO Journal* **2017**, *36*, 3650–3665.
4. Xu, W.; Beebe, K.; Chavez, J.D.; Boysen, M.; Lu, Y.Y.; Zuehlke, A.D.; Keramisanou, D.; Trepel, J.B.; Prodromou, C.; Mayer, M.P.; et al. Hsp90 middle domain phosphorylation initiates a complex conformational program to recruit the ATPase-stimulating cochaperone Aha1. *Nature Communications* **2019**, *10*.
5. Ohashi, T.; Erickson, H.P. Fibronectin aggregation and assembly: The unfolding of the second fibronectin type III domain. *Journal of Biological Chemistry* **2011**, *286*, 39188–39199.
6. Prince, T.L.; Kijima, T.; Tatokoro, M.; Lee, S.; Tsutsumi, S.; Yim, K.; Rivas, C.; Alarcon, S.; Schwartz, H.; Khamit-Kush, K.; et al. Client proteins and small molecule inhibitors display distinct binding preferences for constitutive and stress-induced hsp90 isoforms and their conformationally restricted mutants. *PLoS ONE* **2015**, *10*.
7. Shyu, Y.J.; Liu, H.; Deng, X.; Hu, C.D. Identification of new fluorescent protein fragments for bimolecular fluorescence complementation analysis under physiological conditions. *BioTechniques* **2006**, *40*, 61–66.
8. Picard, D.; Suslova, E.; Briand, P.A. 2-color photobleaching experiments reveal distinct intracellular dynamics of two components of the Hsp90 complex. *Experimental Cell Research* **2006**, *312*, 3949–3958.

© 2020 by the authors. Licensee MDPI, Basel, Switzerland. This article is an open access article distributed under the terms and conditions of the Creative Commons Attribution (CC BY) license (http://creativecommons.org/licenses/by/4.0/).
